# Using Kahoot! as a formative assessment tool in medical education: a phenomenological study

**DOI:** 10.1186/s12909-019-1658-z

**Published:** 2019-06-25

**Authors:** Muhd Al-Aarifin Ismail, Anisa Ahmad, Jamilah Al-Muhammady Mohammad, Nik Mohd Rizal Mohd Fakri, Mohd Zarawi Mat Nor, Mohamad Najib Mat Pa

**Affiliations:** Department of Medical Education, School of Medical Sciences, Universiti Sains Malaysia, Health Campus, Kota Bharu, Malaysia

**Keywords:** Formative assessment, Kahoot!, Gamification, Medical students, Fun learning, Game-based learning

## Abstract

**Background:**

Gamification is an increasingly common phenomenon in education. It is a technique to facilitate formative assessment and to promote student learning. It has been shown to be more effective than traditional methods. This phenomenological study was conducted to explore the advantages of gamification through the use of the Kahoot! platform for formative assessment in medical education.

**Methods:**

This study employed a phenomenological design. Five focus groups were conducted with medical students who had participated in several Kahoot! sessions.

**Results:**

Thirty-six categories and nine sub-themes emerged from the focus group discussions. They were grouped into three themes: attractive learning tool, learning guidance and source of motivation.

**Conclusions:**

The results suggest that Kahoot! sessions motivate students to study, to determine the subject matter that needs to be studied and to be aware of what they have learned. Thus, the platform is a promising tool for formative assessment in medical education.

**Electronic supplementary material:**

The online version of this article (10.1186/s12909-019-1658-z) contains supplementary material, which is available to authorized users.

## Background

Assessment refers to a judgment about the performance of learners on the basis of specific weighted set goals [1]. There are two types of assessment: summative and formative. Summative assessment occurs after instruction and requires that judgments be made about the learning that has occurred. Formative assessment is designed specifically for providing feedback on performance to improve and to accelerate learning [1]. Formative assessment, the assessment of learning, is increasingly being emphasized in academia. It should be seen as an important element in the facilitation of the learning process [3, 4]. Formative assessment is most effective when it is embedded in the teaching and learning activities to facilitate the provision of ongoing timely, specific and actionable feedback to learners [2]. Therefore, formative assessment should be designed to improve students’ understanding of the subject matter.

Formative assessment can be performed in many ways, including paper-and-pencil tests or online quizzes [3]. The fourth industrial evolution has given rise to new methods. Online platforms, such as Kahoot!, Quizziz, Quizlet and Socrative, that apply game-based learning theories have become more common in education. The platforms are widely used as formative assessment tools to promote student learning [4].

Studies have demonstrated the benefits of game-based learning over traditional methods [5, 6]. Game-based learning has been shown to result in significantly improved student performance [6] because of the promotion of learning [7]. Game-based learning increases student motivation [6, 8], increases engagement [9, 10] and provides effective feedback [11].

Kahoot!, a real-time platform for game-based learning, is a free formative assessment tool that has been widely used in education. It has been reported to have more than 30 million users worldwide [12]. Kahoot! allows teachers to create four different types of game-based which are quizzes, surveys, jumbles and discussions in which the participants compete against one another. The top scorers for each question are revealed, and the overall winner(s) is/are displayed on a scoreboard at the end of the session [13].

At the Universiti Sains Malaysia (USM) School of Medical Sciences, formative assessment has been conducted via the e-learning quiz platform eQuiz. The platform has been used to provide feedback to students. The use of eQuiz is not compulsory, and the scores do not contribute to the summative assessments of the students. The data that were previously captured by the learning management system indicate that more than 50% of the students did not participate in the formative assessment activities. To overcome this issue, Kahoot! was introduced as an additional formative assessment tool.

**Fig. 1:**
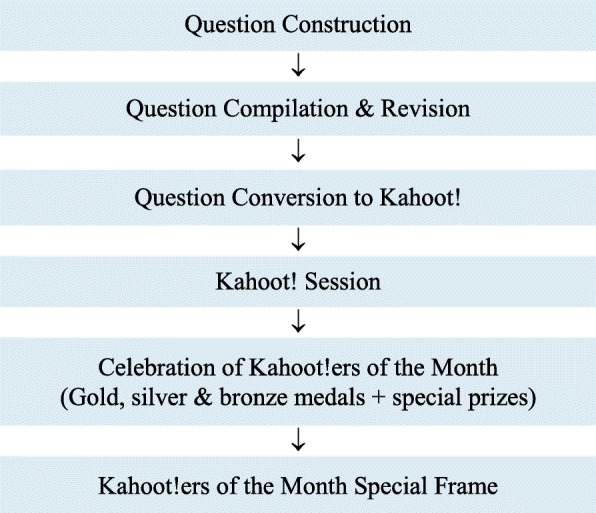
Kahoot! session flow

In the USM School of Medical Sciences, courses are grouped into sequences. Two formative assessment sessions are allocated for each course. The questions to be accessed through Kahoot! are first constructed by the lecturers who are involved in the courses, which span disciplines, such as anatomy, physiology and biochemistry. The questions are submitted to the course coordinators before being converted to the Kahoot! quiz format (Fig. 1). Approximately 120–150 students play the Kahoot! ‘game’ in a typical session. The sessions are conducted by the course coordinators, who are assisted by the e-learning committee members. Each session comprises 20–25 questions. The top three Kahoot! winners get special prizes and medals, and their photographs are displayed on a dedicated frame labelled ‘Kahoot!ers of the Month’ outside their lecture halls.

A quantitative study was conducted to evaluate the students’ perceptions of a Kahoot! session [4]. Kahoot! was found to motivate students and to make learning enjoyable. This qualitative study was conducted to gain an in-depth understanding of the influence of Kahoot! on student learning. Therefore, the current study addressed the benefits of using Kahoot! as a formative assessment tool in medical education.

## Methods

### Research design, setting and sample

The current qualitative study employed a phenomenological design in which purposive sampling was used [5]. Focus group discussions (FGDs) were conducted to explore the medical students’ experiences in the Kahoot! sessions in the USM School of Medical Sciences. The participants were pre-clinical USM medical students who had participated in at least three Kahoot! sessions. Maximal variation sampling was performed to select the participants on the basis of age, gender and ethnicity. The saturation concept was used to estimate the sample size [6]. The participants were invited through a problem-based learning (PBL) group. They were briefed on the study background, protocol and consent form. They were also informed that their identities would be kept anonymous and that they could withdraw from the study at any time. Only the students who gave consent were enrolled in the study. The times and locations of the FGDs were arranged according to the students’ convenience.

### Data collection and analysis

The FGDs were conducted by researchers with expertise in qualitative methods. Bracketing was employed throughout the data collection and analysis [7]. To standardize the data, a four-part interview protocol was developed. It comprised the introduction, trigger questions, probing questions and conclusion (Additional file 1: Appendix A). Each session began with an introduction that explained the ground rules and the FGD process. The sessions began with the open-ended statement: *‘In my experience, the Kahoot! sessions are….’* Researcher bracketing allowed the participants to describe their experiences without contamination by the interviewer [7]. The FGDs were audio recorded and transcribed verbatim. Data collection was stopped once saturation was achieved [8]. Field notes were taken during the interviews. Each FGD lasted approximately 30–45 min. After the first FGD was completed, the data were analysed by the use of ATLAS.ti 7 software. The three steps in the procedure were (i) managing the data, (ii) understanding the data and (iii) interpreting the data.

#### Managing the data

The first step was the verbatim transcriptions of the audio recording of the interview. Each transcript was labelled with a specific code, e.g. FGD [1] 01/06/18. The ‘ [1]’ indicates that this was the first FGD, and ‘01/06/18’ refers to the date of the interview (1st June 2018).

#### Understanding the data

Each sentence of the verbatim text was read very carefully to discern the concepts, ideas and terminology that related to the research objectives. The relevant information was selected on the basis of the research objectives. All of the researchers were involved in this step.

#### Interpretation of the data

This step was executed simultaneously with the second step. The data were coded by the use of thematic analysis [9]. ATLAS.ti 7 software was used for this process [10]. From the verbatim texts, the initial codes were generated; next were the categories, subthemes and themes. Thematic maps generated from the software were reviewed to discern the relationships among the themes. To ensure the credibility, transferability, confirmability and dependability of the data, several measures were taken to enhance the validity of the findings. Table 1 summarizes the trustworthiness criteria that were applied to ensure the rigour of the qualitative research findings [11].

**Table 1:** Trustworthiness criteria ensure the trustworthiness of qualitative findings in this study

Credibility (internal validity)	Transferability (external validity)	Dependability (reliability)	Confirmability (objectivity)
• Prolonged engagement • Data source triangulation • Iterative questioning • Peer debriefing session • Peer scrutiny of findings • Trained moderator • Structural coherence	• Nominated sample • Thick description	• Code–recode procedure	• Unbiased moderator • Audit trail • Analyst triangulation • Reflective analysis

## Results

### Participants’ profiles

A total of 36 students participated in five FGDs in which saturation point was reached during the third session. However, the fourth and fifth FGDs were conducted to confirm the saturation point was indeed reached. The complete demographic profiles of the participants are presented in Table 2.

**Table 2:** Demographic profiles of the participants

Participant characteristics		Number of participants
Gender	Male	10
	Female	26
Ethnic group	Malay	24
	Chinese	4
	Indian	8
Year of study	Year 1	30
	Year 2	6

#### Advantages of Kahoot!

Thirty-six categories and nine subthemes emerged from the FGDs. The subthemes were grouped into three themes: 1) *attractive learning tool*, 2) *source of motivation* and 3) *learning guidance*.

##### Theme 1: attractive learning tool

The participants perceived Kahoot! as a user-friendly platform that makes learning more enjoyable. They also indicated that it promotes active learning (Table 3). The participants found Kahoot! to be more mobile-friendly than eQuiz. In addition, they indicated that the Kahoot! sessions encouraged participation because of the rewards given to the winners at the end of each session. Although participation was not compulsory, the students in the study, all of whom had used this formative assessment tool, stated that ‘all students get involved’ with the activity.

**Table 3:** Theme 1– Attractive learning tool

Subthemes	Categories	Quotations
User-friendliness	Accessibility	*‘It’s easier to use a mobile phone [*for formative assessment with Kahoot! than*] with eQuiz, which requires a [*desktop*] computer or laptop. Kahoot! is better because we can use our mobile phones to get access to the formative assessment. It is easier.’* [P7] FGD [1] 24/01/18
	Specific time & location	*‘… so, when it comes to Kahoot! and what you said, because all of us are in the lecture hall and all of us are answering it together, you have to answer. So, you will answer the Kahoot! questions and actually learn something.’* [P8] FGD [2] 30/01/18
	Student participation	*‘For me, the main advantage of Kahoot! is … all students get involved [*with the activity*].’* [P24] FGD [4] 03/02/18
Fun learning	Gamification	*‘When we play Kahoot!, we feel like we are playing something. We like it because we are in a stressful environment for the entire day, but when we have Kahoot! sessions, we see it as a game, sort of entertainment. At the same time, we get knowledge.’* [P21] FGD [3] 04/01/18
	Audio-visual stimuli	*‘Kahoot! is more interesting because it is colourful. Sometimes there are pictures,* etc.*’* [P25] FGD [4] 03/02/18
	Challenge	*‘… and another thing is Kahoot! is more challenging because the time is like … very short. We have 20 s for each question.…’* [P29–35] FGD [5] 08/02/18
	Fun activity	*‘The Kahoot! session is something like a class … we just go there … attend the session … answer the questions and then we can come out happy.…’* [P21] FGD [3] 04/01/18
	Group learning	*‘When we play Kahoot! … some of my friends might get frustrated. We can help them…. It is helpful….’* [P21] FGD [3] 04/01/18
	Real-time results	*‘We feel really excited … fun … because we can see [*the result*] live [*on the screen*].’* [P15–21] FGD [3] 4/01/18
	Differences in technique	*‘[*Compared to eQuiz*] Kahoot! is much better because the same [*kinds of*] questions are used but with a different technique. It is better.…’* [P1] FGD [1] 24/01/18
Active learning	Promotion of student participation	*‘For me, the main advantage of Kahoot! is everybody gets involved….’* [P28] FGD [4] 03/02/18
	Interactivity	*‘What is good about Kahoot! [*compared to eQuiz*], Kahoot! is more interactive….’* [P3] FGD [1] 24/01/18
	Promotion of active participation	*‘… it requires more focus, active participation during the session….’* [P2] FGD [1] 24/01/18

The participants mentioned that the gamification features in Kahoot! promoted fun learning. They perceived it *‘as a game, sort of entertainment’* ([P21] FGD [3] 04/01/18)*.* The platform was also perceived as challenging and capable of stimulating learning through the use of audio-visual stimuli. The students enjoyed the large group activities because they could compare their performance with that of their peers in real time.

The Kahoot! sessions were perceived as being different and more interesting than the quizzes in the e-learning portal. This finding was supported by the fact that the participants stated that Kahoot! was more interactive, thereby requiring the keener focus and active participation of the students.

##### Theme 2: source of motivation

The participants considered the Kahoot! sessions motivational (Table 4). The sessions promoted continual learning, review and deep learning. After participating in multiple Kahoot! sessions, the participants stated that the platform would help them to improve their academic performance. The Kahoot! sessions increased their motivation through competition, which increased their self-confidence and stimulated their participation in more sessions. Interestingly, the failure to answer questions correctly was not demotivating. One participant stated, *‘It will trigger you to work more’* ([P15] FGD [1] 01/06/18)*.* The participants also perceived the Kahoot! sessions as satisfying and gratifying: *‘When our names are displayed on the screen, we feel very proud, especially when we are in the top three. Our names are also displayed on the frame’* (FGD [4] 03/02/18).

**Table 4:** Theme 2 – Source of motivation

Subthemes	Categories	*Quotations*
Drives learning	Stimulation of continual learning	*‘For me, the Kahoot! session is helpful because of its competitiveness. Psychologically, when there is a competition, we will get ready for it. So, it will encourage us to continuously learn.’* [P3] FGD [1] 24/01/18
	Facilitation of review of subject matter	*‘I used to think that, okay, this question is not that important, so just go through. I don’t go in deep. So, when they ask during Kahoot!, I’ll think … oh, this is important. There is a chance for them to ask this kind of question. So, I go back to my room and study.’* [P5] FGD [1] 24/01/18
	Promotion of deep learning	*‘Kahoot! triggers us to cover the topics properly.’* [P25] FGD [4] 03/02/18
	Perceptions of improved performance	*‘We cover everything so that we can answer Kahoot! properly. So, this automatically improves our academic performance.’* [P4] FGD [1] 24/01/18
	Sense of competition	*‘When we play it together, it’s really fun. When we play it, we know our ranking. If we notice our ranking is at the bottom … [*we will think*] after this: I will focus … must answer the questions correctly. With the usual assessment, we do not know [*immediately whether our answer is correct*] … One more thing: At the end of the session, there are prizes for the winners. So, this will motivate us more….’* [P26] FGD [4] 03/02/18
	Increased self-confidence	*‘Kahoot! always encourages us by telling us* “*You are almost there….” … “Try harder.…” It’s motivating us….’* [P15] FGD [1] 01/06/18
	Motivation to improve performance	*‘When you fail something, it will trigger you to work more and to get it right….’* [P15] FGD [1] 01/06/18
Provides sense of satisfaction	Satisfaction	*‘When our names are displayed [*on the Kahoot!ers of the month frame*], we feel very proud … It is worth what we have [*gone through to*] learn.’* [P20] FGD [3] 04/01/18
	Gratification	*‘When our name is displayed on the screen, we feel very proud, especially when we are in the top three. Our name is also displayed on the frame.’* [P24] FGD [4] 03/02/18

##### Theme 3: learning guidance

The participants believed that the Kahoot! sessions provided learning guidance. They stated that the Kahoot! sessions offered opportunities for feedback, self-reflection and self-assessment. The sessions helped them to increase their understanding of the subject matter and prepare for examinations; thus, their studying was more focused (Table 5). The Kahoot! platform was perceived as an effective tool for assessment and feedback. Furthermore, the sessions encouraged the students to study harder. A participant mentioned the *‘need to study more’* ([P20] FGD [3] 04/01/18)*.* The real-time response in Kahoot! made it interactive; thus, the students received instant feedback that allowed them to know which questions they had answered correctly.

**Table 5:** Theme 3 – Learning guidance

Subthemes	Categories	Quotations
Provides feedback	Insights into areas for improvement	*‘It tells us, as an indicator, we are still not prepared [*on certain topics*]. So, we need to study more.’* [P20] FGD [3] 04/01/18
	Immediate feedback	*‘When we answer Kahoot!, we will know which questions we have answered wrong on the spot.’* [P35] FGD [5] 08/02/18
Encourages self-reflection and self-assessment	Focused learning	*‘By using Kahoot!, I feel it can give me some guidance regarding what is important to study.’* [P2] FGD [1] 24/01/18
	Stimulation of self-reflection	*‘It is like telling us … you are still not ready. You need to study more.’* [P27] FGD [4] 03/02/18
	Top scorers as benchmarks	*‘We will know who the outstanding students are, and we can learn from them.’* [P21] FGD [3] 04/01/18
	Support for weak students	*‘Some of our colleagues may look depressed and frustrated, so we can help them.’* [P20] FGD [3] 04/01/18
	Knowledge testing	*‘Kahoot! really tests our understanding of subjects.’* [P34] FGD [5] 08/02/18
	Assessment of understanding	*‘… you would know your level of understanding [*on the subject*].’* [P34] FGD [5] 08/02/18
Improve understanding	Increased understanding	*‘… lecture note we read. Okay, we know these are the symptoms of it. Then suddenly we see in Kahoot! two or three, a few more symptoms … “Ooh, these are also the symptoms of the disease.” Then we get the knowledge.’* [P21] FGD [3] 04/01/18
Examination preparation	Test of ability to respond quickly	*‘Kahoot! also tests a speedy response because we have only a few seconds to answer each question.’* [P34] FGD [5] 08/02/18
	Development of rapid information processing	*‘It trains us to think fast. It gives us limited time to think. It will train our minds to answer questions fast….’* [P21] FGD [3] 04/01/18
	Early exposure to MCQs	*‘I think that would be particularly helpful, especially in [*answering*] MCQs….’* [P21] FGD [3] 04/01/18
	Promotion of examination preparation	*‘We will make better preparations….’* [P25] FGD [4] 03/02/18
Focused learning	Guidance in setting learning priorities	*‘… we can see which topics are important … which topics are less important, which topics we have studied, which ones we have not.…’* [P21] FGD [3] 04/01/18
	Promotion of goal setting	*‘With Kahoot!, we can have our own target … like, this time I study this much, so my target should be this much.’* [P17] FGD [3] 4/01/18

The participants stated that the Kahoot! sessions provided guidance on the content to be learned and that it encouraged self-reflection. The feature of displaying the top scorers in real time provided ‘benchmark peers’, and this helped the low academic achievers to seek necessary support from their peers and lecturers. The Kahoot! sessions also tested their understanding of the material, thus helping them to improve their academic performance.

Regarding examination preparation, a unique feature of the platform is that besides giving correct answers, the speed of response is also important for the students to score more points during Kahoot! sessions. The participants mentioned that this feature trained them to think fast not only for examinations but also for their lives as future medical practitioners. Early exposure to multiple-choice questions (MCQs) and thus the examination was another advantage of the Kahoot! sessions.

The students needed to prioritise the topics to be studied. They indicated that after completing several Kahoot! sessions, they had a clearer idea of the important topics to be studied in deep. A participant said, *‘We can see which topics are important … which topics are less important’* ([P21] FGD [3] 04/01/18).

## Discussion

Good formative assessments strengthen the students’ capacity to regulate their own performance. Nicol and Macfarlane-Dick [12] identified seven principles of good feedback practices. Good feedback practices 1) help to define good performance, 2) facilitate self-reflection; 3) deliver high-quality information to students about their learning, 4) encourage teacher and peer dialogue, 5) promote positive motivational beliefs and self-esteem, 6) close the gap between current and desired performance and 7) provide instructors with information that can be used to shape their teaching. Regarding formative assessment, the study showed that the students perceived the Kahoot! sessions as an effective feedback tool. It is an attractive learning tool that is a source of motivation and guidance for student learning.

### Attractive learning tool

Kahoot! was perceived as being more user-friendly than eQuiz. This finding was similar to that of a previous previous study [4]. The students can benefit from formative assessment by downloading the Kahoot! application, which is available free in the Play Store (for Google’s Android operating system) and the App Store (for Apple’s iOS operating system). They can participate in the sessions with just a single tap. In contrast, participation in eQuiz involves more steps, such as browsing the e-learning portal, logging in with their user names and passwords and searching for their courses. The multiple steps might explain the students’ preference for Kahoot! as a formative assessment tool.

**Fig. 2:**
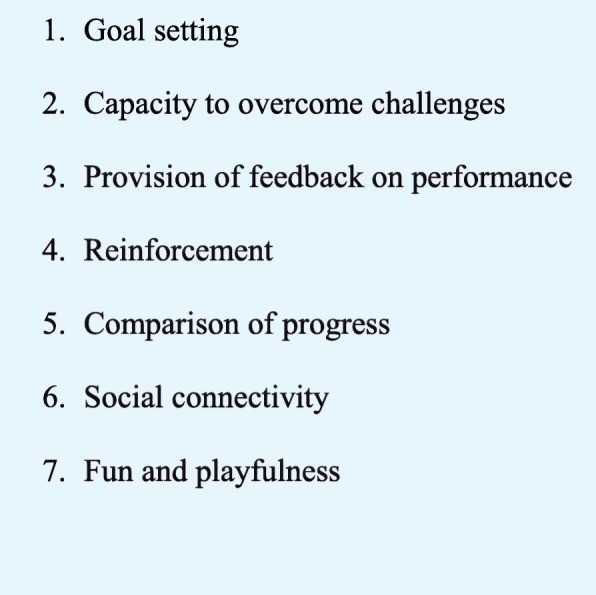
Elements of persuasive architecture of gamification

This study shows that student engagement in formative assessment was higher with Kahoot! than with eQuiz. This was consistent with the previous findings that fewer than 50% of students used eQuiz. After the change to Kahoot!, more than 90% participated in the formative assessment activities. An effective tool for gamifying learning, Kahoot! possesses all seven elements of the persuasive architecture of gamification (Fig. 2; [ [13]. It sets goals, offers challenges and provides feedback. It also provides reinforcement, facilitates learners’ comparisons of their own and their peers’ progress, enables social connectivity and creates a fun learning environment. Learning with Kahoot! was perceived as fun mainly because of the gamification features and the audio-visual stimuli. The platform was also perceived as providing challenging activities, such as games for large groups for which the results are available in real time. A Kahoot! session is different from and more interesting than an eQuiz session. Furthermore, Kahoot!‘s interactive features promote active learning through increased engagement.

Trumpower and Sarwar [14] asserted that user-friendliness is crucial for effective formative assessment. The specificity and accuracy of a platform are of no value if the users (students and teachers) cannot understand how to use it [15]. The current study has demonstrated that Kahoot! is user-friendly; however, a strong internet connection and the appropriate devices must be available before and during the session. For a session to be successful, the faculty members should also be familiar with the platform. However, Kahoot! has some limitations. It is suitable for multiple-choice rather than essay questions.

Medical training is considered to be intense and demanding; therefore, medical students are predisposed to psychological distress [16]. Previous studies have found the worldwide prevalence of psychological distress among medical students to be 21–56% [16]. A fun learning environment has been shown to reduce psychological distress [17]. In addition to having positive effects on education, gamification creates a fun and entertaining environment [18]. The current study shows that the Kahoot! platform is effective at changing a stressful environment into a joyous one. This finding is consistent with that of a previous quantitative study in which students perceived Kahoot! sessions as fun [4]. However, institutions that wish to adopt Kahoot! for fun learning should note that the sessions should be properly planned to ensure that the students achieve the intended outcomes and avoid the excessive fun that could create disastrous teaching environment and lead to negative consequences [19].

#### Sources of motivation

Formative assessment can have a significant effect on student motivation and achievement [20] if it is designed to stimulate the extrinsic aspects of student motivation [21]. Kahoot! has been shown to be an external factor in facilitating continual learning, which could improve academic performance. Its competitive environment is key [22]. Another interesting aspect that has been demonstrated in the current study is the sense of satisfaction experienced by the students. Thus, Kahoot! indirectly engenders positive feelings in students regarding their academic progress [23].

#### Learning guidance

As has been demonstrated in this study, formative assessment is most effective through the ‘catalytic effect’ of specific and actionable feedback to learners [2]. The sessions encouraged the participants to reflect on their strengths and weaknesses. Furthermore, unlike other formative assessment approaches, Kahoot! allows teachers to provide instant feedback. The session allows teachers to interact with their students and to clarify material related to the questions. In addition to facilitating teacher feedback, the sessions also promote self-assessment. An ‘assessment for learning’ tool [24], the Kahoot! session indirectly prepares students for summative assessment by highlighting the important topics to be reviewed. In sum, Kahoot! sessions serve as an assessment of learning.

#### Other advantges of Kahoot!

Kahoot! offers several other advantages. The platform: 1) is free; 2) uses multiple formats, i.e. quizzes, discussion questions or surveys; 3) is user-friendly; 4) facilitates participation through an automatically generated game pin; 5) is compatible with smartphones, tablets and computers; 6) has music and colourful illustrations that generate excitement; and 7) has a flexible and adjustable response time based on question complexity. In addition, instructors can download, review and save the students’ results to analyse student performance [4]. Therefore, it is recommended that medical schools use this free platform as a formative assessment tool to promote learning. Kahoot! can be incorporated into teaching and learning sessions, such as lectures, tutorials and problem-based learning activities, for effective formative feedback.

### Limitations of the study

This qualitative study was conducted in a single institution; therefore, caution should be observed in extending the findings to other populations. The study found that students perceived Kahoot! as a useful tool for formative assessment. They indicated that participation in Kahoot! sessions can improve their academic performance. However, further study is need to determine the effects of Kahoot! on academic performance.

## Conclusions

Kahoot! is an innovative formative assessment tool. This study suggests that Kahoot! sessions motivate students to study, focus on the important concepts and reflect on what they have learned. Therefore, this platform holds promise for facilitating formative assessment in medical education. Health professions educators are therefore encouraged to incorporate Kahoot! into their teaching and learning activities particularly for formative assessment.

## Additional file

Additional file 1: **Appendix A.** Focus Group Discussion Protocol. (DOCX 18 kb)

## Data Availability

The datasets used and analysed during the current study are available from the corresponding author upon a reasonable request.

## Abbreviations

FGD: Focus group discussion
MCQs: Multiple-choice questions
PBL: Problem-based learning
USM: Universiti Sains Malaysia
