# Supplementary material for: Using Kahoot! as a formative assessment tool in medical education: a phenomenological study
Source: BMC Med Educ. 2019 Jun 25;19:230. doi: 10.1186/s12909-019-1658-z (PMC6593549; doi:10.1186/s12909-019-1658-z)
Supplement: Supplementary file 1 — Appendix A. Focus Group Discussion Protocol. (DOCX 18 kb) [file 12909_2019_1658_MOESM1_ESM.docx]

Appendix A: **Focus Group Discussion Protocol**

**Introduction:**

1. Welcome

Introduce yourself and the note-taker. Circulate the Participant Information Sheet with a few quick demographic questions (gender, ethnic group and year of study) while you are introducing the focus group.

2. Explanation of the process

Ask the group whether anyone has previously participated in a focus group. Explain that the use of focus groups in health and human services research has been increasing.

*About focus groups*

• We learn from you (positive and negative).

• Not trying to achieve consensus; we are gathering information.

• No virtue in long lists; we are looking for priorities.

• In this project, we are conducting focus group discussions to gather in-depth information from a small group of people. This allows us to understand the context and helps us to explore topics in greater detail than is possible in a written survey.

*Logistics*

• Focus group will last approximately 45–90 minutes

• Feel free to move around

• Where is the bathroom? Exit?

• Help yourself to refreshments

3. Ground rules

Ask the group to suggest some ground rules. After the participants brainstorm, make sure the following are on the list.

• Everyone should participate.

• Information provided in the focus group must be kept confidential.

• Stay with the group and do not engage in side conversations.

• Turn off cell phones if possible.

4. Turn on tape recorder

5. Ask the group whether there are any questions before we get started, and address those questions.

6. Introductions

Go around the table; ask participants where they were born.

*Discussion begins. Be sure to give the participants time to think before they answer the questions. Don’t move too quickly. Use the probes to ensure that all issues are addressed, but move on when you feel that the information is becoming repetitive.*

7. The checklist for the FGDs are as follows:

| To-do list |  |
| --- | --- |
| 1. Participant information sheet and consent forms (one copy for participants, one copy for the team) |  |
| 1. Evaluation sheets: one for each participant; name tents, pads and pencils for each participant |  |
| 1. Focus group discussion guide for facilitator |  |
| 1. 2 (TWO) recording devices |  |
| 1. Batteries for recording devices |  |
| 1. Extra tapes for recording devices |  |
| 1. Permanent marker for indicating FGD name, facility, and date on tapes |  |
| 1. Notebook for note taking |  |

**Focus Group Discussion Guide**

| Interview Guide | Researcher remarks | Researcher Comments/ Reflections |
| --- | --- | --- |
| Part 1: Introduction  Researcher to   - - 1. introduce him/herself     2. thank students for their participation in this study     3. explain the purpose and format of the interview     4. obtain permission to make audio recording     5. provide reassurance about confidentiality   * explain terminology if requested   - - - 1. Formative assessment       2. eQuiz and e-learning |  |  |
| Part 2: Trigger questions  Researcher to say:   - - *‘Please continue this sentence: “To me, Kahoot! is …”’*   - *‘You have experienced several Kahoot sessions, please share your experiences.’*   - *‘Tell me anything you know about Kahoot.’*   - *‘Tell me about the benefits that you have gained during and after Kahoot sessions.’* |  |  |
| Part 3: Probing Questions  Researcher to say:   - - *‘Please elaborate on your point on …’*   - *‘Can anyone share a similar experience regarding …’*   - *Does anyone have a different experience regarding …’*   - *Any other additional information regarding your experiences during the Kahoot sessions?* |  |  |
| Part 4: Conclusion  Researcher to:   - - ask participants to share their thoughts if they so desire   - give the participants opportunities to discuss any other issues regarding the topic   - express his/her appreciation to all the participants   - end the session |  |  |
